# Policies on children and schools during the SARS-CoV-2 pandemic in Western Europe

**DOI:** 10.3389/fpubh.2023.1175444

**Published:** 2023-07-25

**Authors:** Antoni Soriano-Arandes, Ana Brett, Danilo Buonsenso, Louise Emilsson, Isabel de la Fuente Garcia, Despoina Gkentzi, Otto Helve, Kasper P. Kepp, Maria Mossberg, Taulant Muka, Alasdair Munro, Cihan Papan, Aida Perramon-Malavez, Frederik Schaltz-Buchholzer, Pierre R. Smeesters, Petra Zimmermann

**Affiliations:** ^1^Pediatric Infectious Diseases and Immunodeficiencies Unit, Hospital Universitari Vall d'Hebron, Barcelona, Spain; ^2^Infectious Diseases Unit and Emergency Service, Hospital Pediátrico, Centro Hospitalar e Universitário de Coimbra, Coimbra, Portugal; ^3^Department of Woman and Child Health and Public Health, Fondazione Policlinico Universitario A. Gemelli IRCCS, Milan, Italy; ^4^Department of Medical Epidemiology and Biostatistics, Karolinska Institute, Solna, Sweden; ^5^Department of General Practice, Institute of Health and Society, University of Oslo, Oslo, Norway; ^6^Pediatric Infectious Diseases, National Pediatric Center, Centre Hospitalier de Luxembourg, Luxembourg, Luxembourg; ^7^Department of Paediatrics, Patras Medical School, Patras, Greece; ^8^Department of Health Security, Institute for Health and Welfare, Helsinki, Finland; ^9^Pediatric Research Center, Children's Hospital, Helsinki University Hospital, University of Helsinki, Helsinki, Finland; ^10^Section of Biophysical and Biomedicinal Chemistry, DTU Chemistry, Technical University of Denmark, Kongens Lyngby, Denmark; ^11^Department of Pediatrics, Clinical Sciences Lund, Lund University, Lund, Sweden; ^12^Institute of Social and Preventive Medicine, University of Bern, Bern, Switzerland; ^13^Epistudia, Bern, Switzerland; ^14^NIHR Southampton Clinical Research Facility and Biomedical Research Centre, University Hospital Southampton NHS Foundation Trust, Southampton, United Kingdom; ^15^Faculty of Medicine, Institute of Life Sciences, University of Southampton, Southampton, United Kingdom; ^16^Institute for Hygiene and Public Health, University Hospital Bonn, Bonn, Germany; ^17^Computational Biology and Complex Systems (BIOCOM-SC) Group, Department of Physics, Universitat Politècnica de Catalunya (UPC·BarcelonaTech), Barcelona, Spain; ^18^Bandim Health Project, Department of Clinical Research, University of Southern Denmark, Odense, Denmark; ^19^Department of Pediatrics, University Hospital Brussels, Academic Children’s Hospital Queen Fabiola, Université Libre de Bruxelles, Brussels, Belgium; ^20^Molecular Bacteriology Laboratory, Université Libre de Bruxelles, Brussels, Belgium; ^21^Department of Community Health, Faculty of Science and Medicine, University of Fribourg, Fribourg, Switzerland; ^22^Department of Paediatrics, Fribourg Hospital, Fribourg, Switzerland

**Keywords:** COVID-19, children, mitigation, masks, vaccination, school closure, testing, ventilation

## Abstract

During the pandemic caused by severe acute respiratory syndrome coronavirus 2 (SARS-CoV-2), mitigation policies for children have been a topic of considerable uncertainty and debate. Although some children have co-morbidities which increase their risk for severe coronavirus disease (COVID-19), and complications such as multisystem inflammatory syndrome and long COVID, most children only get mild COVID-19. On the other hand, consistent evidence shows that mass mitigation measures had enormous adverse impacts on children. A central question can thus be posed: What amount of mitigation should children bear, in response to a disease that is disproportionally affecting older people? In this review, we analyze the distinct child versus adult epidemiology, policies, mitigation trade-offs and outcomes in children in Western Europe. The highly heterogenous European policies applied to children compared to adults did not lead to significant measurable differences in outcomes. Remarkably, the relative epidemiological importance of transmission from school-age children to other age groups remains uncertain, with current evidence suggesting that schools often follow, rather than lead, community transmission. Important learning points for future pandemics are summarized.

## 1. Introduction

During the pandemic caused by severe acute respiratory syndrome coronavirus 2 (SARS-CoV-2) (1), mitigation policies in relation to children have been a topic of considerable uncertainty and debate, most importantly because of the distinct disease manifestations, transmission dynamics, and public health trade-offs in children compared to adults (2–4).

**Figure 1:**
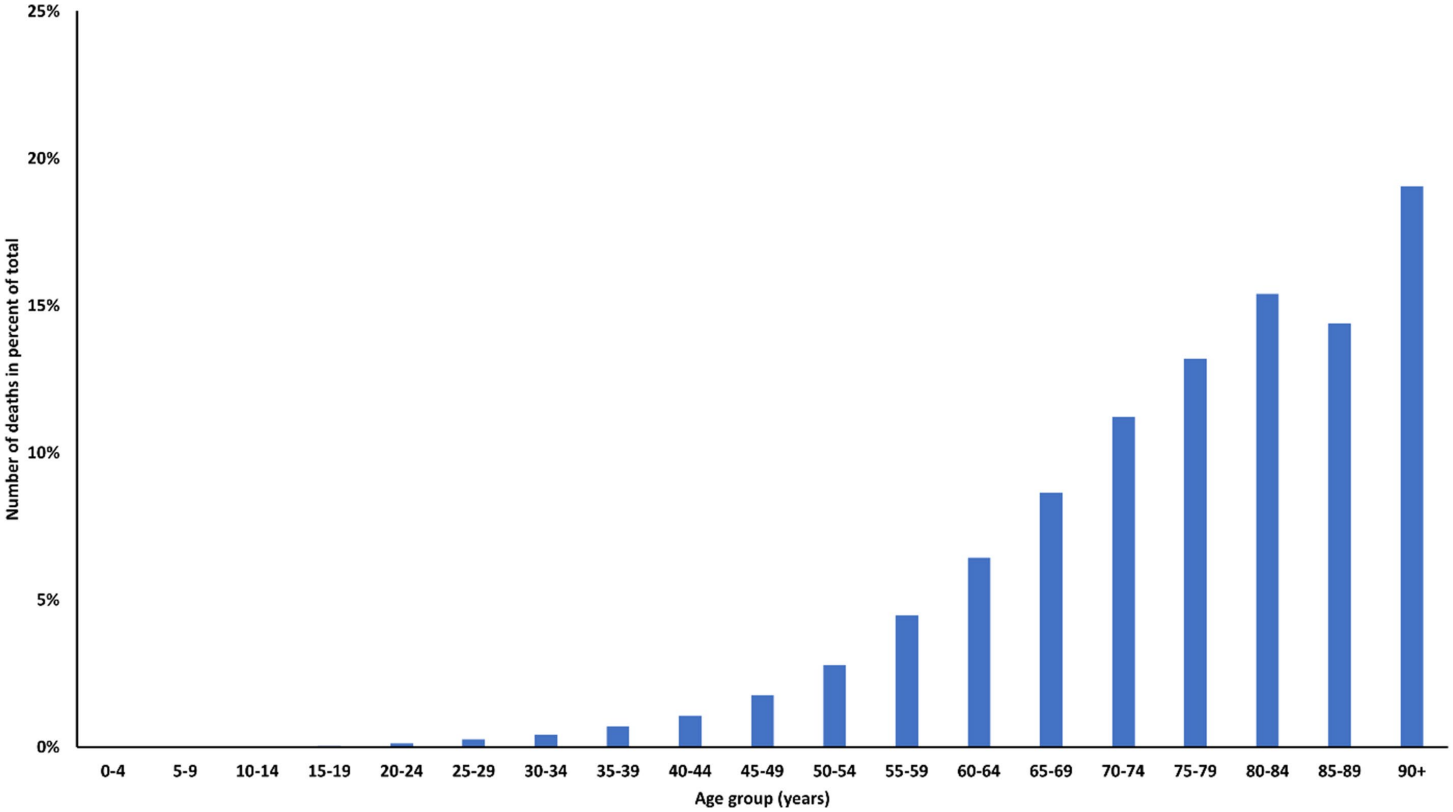
Percent of total COVID-19 registered deaths assigned to age groups, UNICEF/MPI DR COVerAGE database.

As of January 2023, the COVerAGE database from UNICEF reported a total of 4.1 million global deaths due to coronavirus disease (COVID-19). Children and adolescents under 20 years of age accounted for 0.4% (5, 6) of these deaths, despite representing 30% of the world population (7). In high-income countries, the same age group accounted for 22% of the population, 23% of COVID-19 cases, but only 0.1% of registered COVID-19 deaths (8). Potential reasons for the lower proportion of COVID-19 deaths in children in high-income countries are (i) the older mean population age in high-income countries, which is associated with a steep rise in hospitalization and mortality rates due to COVID-19 (Figure 1) (9); (ii) a lower proportion of children with co-morbidities associated with more severe COVID-19, and (iii) differences in the coding of COVID-19 deaths.

Some children are at higher risk for severe COVID-19, and protection from multisystem inflammatory syndrome in children (MIS-C) associated with COVID-19, as well as long COVID is important (10, 11). Moreover, if children contribute substantially to transmission, epidemiological restrictions may be justified beyond individual disease risk (12). However, as reviewed below, uncertainty remains on the importance of transmission dynamics between school-age children and older and vulnerable people, with whom they tend to have less contact (13) and consequently transmit less to, at least in comparison to same-age individuals (14–17).

These data pose a dilemma between epidemiological control versus the individual benefit-harm ratio which is central to medicine and public health (first, do no harm): what amount of mitigation should children bear, in response to a disease that is disproportionally affecting older people (5)? Mitigation measures had an extremely large impact on children, especially school closures, interruption of co-ordinated sport and other free-time activities and disruption of other public health efforts (18, 19). Damages arising from school closures include education loss, lack of social interaction, loss of indirect benefits such as meals, as well as marginalization and inequality (20, 21). A recent meta-analysis showed substantial overall learning deficit, which arose early in the pandemic and persists over time, affecting disproportionally children from low socio-economic backgrounds (22). This adds to the “education emergency of unprecedented scale” (19). A massive loss of nutritional support has been reported globally, with UNICEF estimating that, already during the first year of the pandemic, 370 million children in 150 countries missed school meals (23). Furthermore, some morbidities, that are also associated with an increased risk for severe COVID-19 or potential sequelae, such as being overweight, diabetes, and mental health problems, increased in children due to interventions aimed to reduce SARS-CoV-2 transmission (24–26). Medical care (including diagnosis, treatment and psychosocial support) of patients, including children, with chronic diseases or cancer, has been negatively affected by the pandemic (27–29).

European countries took reasonably similar approaches for the adult population when the pandemic began in 2020, but remarkably diverse strategies were implemented when it came to children. This included major differences in school closure policies (30), social and educational options offered to children during the crisis, as well as general mitigation (e.g., mask use) and pediatric vaccination policies. For example, Germany and Italy applied authoritative policies with strict non-pharmaceutical interventions (NPIs), arguably restricting children as much or even more than adults at certain times. In contrast, Scandinavian countries never imposed mask use on primary school (defined here as primary level education for those up to 12 years of age) children and avoided strict NPIs such as curfews, giving children more options to socialize within the community (31). Vaccination or testing was also not a requirement for attending school, although vaccination was recommended and testing strongly recommended during the early Omicron wave in Denmark, before abandoning all mitigations on February 1, 2022.

The division between these two strategies of pediatric mitigation, a conservative approach emphasizing epidemiological control versus a strategy emphasizing approaches with many public health trade-offs, is probably the most defining feature of the Western European pandemic responses. Understanding the origin of these differences, the debates taking place behind them, and their benefits and harms, seems to be one of the most essential learning points for future pandemics. To help such a discussion, this review analyses the epidemiology, policies, trade-offs, and outcomes of the COVID-19 pandemic in children in Western Europe.

## 2. Methodology

Given the complexity of the topic, the heterogeneity and poor quality of studies in certain areas, as well as the large number of social and mitigation factors affecting these analyses, a systematic review was not deemed as feasible. A narrative review with the main aim of identifying key aspects that should be considered moving forward was judged as more appropriate. In February 2023, we searched PubMed, Web of Science, Google Scholar, and Cochrane library databases to identify relevant studies related to SARS-CoV-2, children, schools, and mitigation measures. We considered all types of articles, including clinical trials, observational studies, reviews, and meta-analyses. As this is a narrative review, we gave preference to the most recent and least bias-prone studies available in the current literature (ranking observational studies and studies using proxies of infection lower). Nevertheless, uncertainties and disagreements have been carefully discussed throughout the manuscript.

## 3. Epidemiology of pediatric COVID-19 in Europe

In the following chapter, we examine the multifaceted complexities of the COVID-19 pandemic, which encompass variations in susceptibility, transmissibility, exposure to SARS-CoV-2, and severity among children. Furthermore, we explore the impact of pediatric COVID-19 on the European pandemic, with a particular focus on the effect of distinct SARS-CoV-2 variants. Limitations of the available data and testing methods are also thoroughly addressed to provide a comprehensive overview of the current knowledge in the field.

### 3.1. Infection and transmission of SARS-CoV-2 in children in Europe

As a null hypothesis, it could be assumed that SARS-CoV-2 has similar attack rates in children and other population sub-groups. However, population subgroups, including children, had distinct exposure to SARS-CoV-2 due to differences in social contact patterns and NPIs, as well as due to inherent biological heterogeneity in susceptibility and transmissivity (32–34). These heterogeneities varied between countries and complicate epidemiological estimates for children. For example, school and work closures moved exposure into the community and homes for both children and adults in different ways at different times during the pandemic (35).

Biological heterogeneity also affects measurements of exposure. While polymerase chain reaction (PCR) tests have similar sensitivity and specificity for all subgroups, antibody tests require that individuals seroconvert upon exposure. However, some individuals abort infection without developing an antibody response (36–38), plausibly because of an asymptomatic or mild infection, or because of heterogeneous immune responses (39). Moreover, children can mount an immune response to SARS-CoV-2 without virological confirmation of infection; therefore routine virological and serological testing may not identify all exposed children (40). Antibody responses to some extent also correlate with the severity of the infection (41, 42). Children may be less likely to seroconvert or may have lower antibody responses (43–45), likely because children more often clear SARS-CoV-2 infections with innate immunity. Moreover, a fraction of the population does seroconvert but seroreverts before measurement, adding to false negative infection prevalence estimates. Seroreversion may depend on the virus variant and immunity from vaccination or previous infection, including infection with other coronaviruses (46). These limitations make seroprevalence a lower bound for the true prevalence (47, 48). Furthermore, surveys need to be fully population-representative to estimate the true prevalence in a population. Children were tested less compared with adults, especially early in the pandemic (49) and since many children are asymptomatic or have mild disease (50) they might not be tested. However, even when testing is applied systematically, the secondary attack rate depends on age (51).

A meta-analysis of early studies suggested that children are substantially less susceptible to infection with SARS-CoV-2 (52), with the limitations mentioned above regarding representability and false-negative test rates for pediatric compared with adult infections. A recent, detailed social-mixing analysis using PCR data without the seroconversion/−reversion limitations of antibody surveys confirmed that pediatric susceptibility to infection is about half that of adults (53).

The Institute for Health Metrics and Evaluation (IHME) has provided estimates of cumulative country-specific attack rates up to November 2021 (just before the Omicron variant took over) (54), with the caveat that the estimates may be biased toward adult population surveys. Although concerns have been raised about the IHME mortality model which may explain the anomalous IHME infection fatality rates (IFRs) (55), the IHME infection estimates are reasonably consistent with national surveys and take into account seroreversion.

Different responses and social structures could be expected to produce variations in children-to-adult attack rates in various countries, although data on this is limited. When the IHME estimates are used (54), infection levels differ between countries, with relatively lower levels of infection in some Nordic countries such as Norway, Denmark and Finland, and higher levels in Sweden and many countries in continental Europe (54).

Social mixing is very heterogeneous, both across age groups and within age groups. European pre-pandemic contact matrices (13) show the largest mixing between similar age groups. The highest social mixing is seen in adults at working age, whereas older people have the least social contact with children and most contact with other adults or older adults. These data suggest that interactions between children and grandparents, a concern often raised during the pandemic, are fewer than naively assumed in a homogeneously transmitting population, consistent with the view that school transmission reflects community transmission, not the other way around (17, 56). The extent of interactions likely varies between countries and regions, and understanding these differences is crucial for developing effective public health policies (57, 58).

It also remains unclear whether having children at home or in the community rather than in school increases or reduces infection risk. A meta-analysis of 90 studies concluded that “risks of infection among children in educational settings was lower than in communities” (59). However, this may change when social distancing and mitigations are applied in schools.

Even if we were to presume that children were more infected in schools, spill-over to other age groups might still be lower in an open-school context, as the social contact rate to older groups at home or in the community increases with schools closed, which could lead to higher transmission to the vulnerable. Interestingly, a large study performed in Northern California showed that the risk for hospitalization or intensive care admission (ICU) was lower for adults living with young children, suggesting that endemic coronavirus cross-immunity may play a role in protection against severe COVID-19 (60).

With the limitations discussed above and lack of data suggesting otherwise, we will assume that European countries share age-stratified contacts and infection patterns that are roughly heterogeneous in the same way. If so, a country’s pediatric attack rate would be approximately in proportion to the general population infection levels (54) (Table 1, bottom). For example, Germany’s estimated cumulative population infection rate up to November 2021 of 14.4% (54) was consistent with pediatric seroprevalence estimates until March 2021 of 10.8% (61), especially considering the half year gap in the timing of the survey and uncertainties regarding seroreversion. This relative size of the pediatric pandemic may have changed during the pandemic with different virus variants, with the highest infection rates seen for Omicron. However, this effect may also reflect increased application of pediatric testing.

**Table 1:** Pandemic school responses and pediatric disease burden in Western European countries (as per November 2022).

Response	UK*	IRE	GER*	NETH	BEL	LUX	FRA	DEN	NOR	SWE	ICE	FIN	SPA	POR	ITA	SWI*	AUS	GRE
No. weeks with closed schools (UNESCO) until end of 2022	16	16	14	12	9	9	7	8	5	0	0	8	10	12	13	6	15	18
No. weeks with partially-open schools (UNESCO) until end of 2022	11	11	24	19	20	6	5	27	24	24	6	25 (0 for primary school)	5	12	25	0	24	19
End of mandated restrictions at school (date)	Sep 2021	28/02/2022	Not yet in some states	25/04/2022	07/03/2022			01/02/2022	12/02/2022	01/04/2022	25/02/2022	13/01/2022	01/09/2022	01/09/2022	01/09/2022		25/04/2022	
Masks for primary school children	No	No	Yes until April 2022	No	At specific periods	At specific periods	At specific periods	No	No	No	No	National recommendation for ages 12 and over until October 2021	Yes until April 2022	Yes until Sep 2022	Yes until Sept 2022	Yes briefly	Yes until Feb 2022	Yes
Masks for secondary school children	>12y (indoors but outside classroom)	Yes	Yes until April 2022	At specific periods (removed in class)	Yes	Yes until March 2022	Yes	No	Yes	No	No	Yes until October 2021	Yes until April 2022	Yes until Sep 2022	Yes until Sept 2022	Yes	Yes until Feb 2022	Yes
Testing and quarantine rules (pandemic summary until end of 2022)	Test and quarantine until 31 Mar 2022 (test/stay for contacts from Sep 2021)	Test and stay	Yes	Yes	Yes	Yes	Yes	Yes until Oct 2021	Yes	Yes	Yes	Yes until Jan 2022	Yes until Jan 2022	Yes, until Jan 2022	Yes	Yes	Yes	Yes
Asymptomatic testing (pandemic summary until end of 2022)	Secondary schools only—twice weekly from Mar 2021 to Mar 2022	No	Yes	Yes	Yes	Yes (stop in 2022)	Yes	Yes	Yes (twice a week)	No	No	Yes, close contacts, subject to local evaluation (no screenings)	Yes, all contacts to initial case (bubble groups)	Yes, i.e., before starting the 2021–2022 academic year for >13y	Yes	Yes	Yes (mobile teams)	Yes
Infection rate, up to Nov 2021	27.7%	22.3%	14.4%	27.1%	32.0%	24.1%	23.3%	13.5%	11.2%	22.4%	7.9%	8.6%	24.9%	21.9%	19.6%	19.7%	22.4%	15.2%
Deaths 0–9y to April 1, 2022	4.8		4.9	1.7	4.9 (0–24)	0	4.4	4.9		12.3		1.9	5.7	3.4	5.0	3.4	4.6 (0–4)	
Deaths 10–19y to April 12,022	11.0		4.7	3.5	4.9 (0–24)	0	3.4	5.9		6.7		1.6	5.6	2.9	5.1	1.2	5.9 (5–14)	

UNESCO school closure dataset, https://covid19.uis.unesco.org/global-monitoring-school-closures-covid19/country-dashboard/; PH measures at schools by country, https://phsm.euro.who.int/measuresDatabase; Finland, https://thl.fi/en/web/infectious-diseases-and-vaccinations/what-s-new/coronavirus-covid-19-latest-updates/situation-update-on-coronavirus/coronavirus-infections-in-schools; https://www.thl.fi/episeuranta/tautitapaukset/coronamap.html.

*Heterogeneity of policy implementations in different states.

Regarding transmission there appears to be a clear difference between adolescents and young children: in regards to susceptibility, young children’s household secondary attack rate is much lower than that of adolescents (which is similar to adults) (59). This is reflected in prevalence in representative surveys [such as the ONS in the UK (62)], where the prevalence in primary-aged children was significantly lower than in adolescents for almost all of the first 18 months of the pandemic. In contrast, household transmission rates from younger children appears higher than from adolescents (although equal to or lower than from adults) (63, 64). This is likely reflective of the closer contact of caregivers with young children during illness, as viral loads appear lowest in younger children (64, 65). In addition, the age-dependent dynamics of transmission have changed dramatically over the course of the pandemic as result of shifting patterns of immunity. When adults became fully-vaccinated yet children were almost entirely unvaccinated, they had a much higher prevalence. This was the case during the latter Delta period (66) and initial Omicron wave. Now owing to higher rates of infection-induced immunity, younger children have again had the lowest rates of disease prevalence of any age group.

We might expect in the future that these patterns will morph into more similar patterns to those of seasonal respiratory viruses as a susceptible pool of young children keeps being added, and older cohorts keep adding to immunity through repeated infection. This is important for future pandemics. While we model respiratory virus pandemic response on influenza which include a disproportionately large role of young children in transmission; an important cause of this is the differences in immunity. For COVID-19, where there were no differences in immunity, young children did not play a disproportionate role (and if anything, played a disproportionately small role). This makes school closures less effective than might be predicted, impacting the cost–benefit analysis. For future novel pathogens with little or no population immunity, children may not play a disproportionately large role in transmission and school closures may be a less effective means of blunting transmission.

### 3.2. Impact of pediatric COVID-19 during the pandemic in Europe

Estimating the severity of infections requires comparison of hospitalization and death rates, or other adverse outcomes, against an estimate of the real prevalence of the disease in the corresponding age group.

As early as February 2020, a lower susceptibility of children to severe COVID-19 was suggested by data available from China (67). The steep age-related disease risk gradient and relative mild disease in children also quickly became evident during the first wave in Europe (68). Furthermore, seroprevalence studies began to emerge in Spring 2020 and pointed toward substantially higher infection levels and correspondingly lower IFRs than deduced from case fatality rates, e.g., the Danish blood donor study (69, 70). In late April 2020, the Danish *Statens Serum Institut* published data indicating a nearly exponential dependency of mortality with age (71). Such patterns have since been confirmed (72), also in large international studies (73, 74). Symptomatic infection in children and adolescents less than 17 years of age was associated with approximately 1.2% crude hospitalization risk and 0.1% crude risk of ICU admission with population-wide infection-outcome risks being smaller due to additional non-symptomatic cases. These numbers representing 10 EU countries, provide a reasonable indication of the disease impact on children in the first waves of the pandemic in Europe (73). In many countries, hospitalization rates in children infected with respiratory syncytial virus and influenza are much higher than in those infected with SARS-CoV-2 (75–78). With the arrival of Omicron and mass vaccination, the overall IFR fell further and was estimated to be as low as 6.2 per 100,000 people aged 17–72 years in a large collaborative Danish study (79). In addition to those direct short-term risks, there are additional longer-term effects, as discussed below.

### 3.3. MIS-C and long COVID

MIS-C, a disease characterized by hyperinflammation and multi-organ involvement presenting with clinical features similar to Kawasaki disease or toxic shock syndrome, has been observed 2 to 6 weeks after a SARS-CoV-2 infection, mainly in children. Although MIS-C is rare, affecting less than 0.1% of SARS-CoV-2-infected children, it has been associated with high rates of ICU admissions (~80%) and even death (~2%) (80).

The incidence of MIS-C varies between countries, depending on genetic and socio-economic factors, circulating virus variants, infection incidence and population immunity. A total of 111 cases of MIS-C were diagnosed in France from April 2020 to January 2021 (~0.7 cases per 100,000 children during 9 months) (81). Similar numbers were reported from Spain, where 45 children were admitted to ICU with MIS-C from March to June of 2020 (82). The national Swiss Paediatric Surveillance Unit reported that 204 children were hospitalized with MIS-C between March 2020 and March 2022 (13 per 100,000 children). In an international study which included 904 children with MIS-C, a substantial decrease in incidence has been observed throughout the pandemic. It has been hypothesized that this might be due to immunity through previous infection or COVID-19 vaccination or due to viral factors (83). COVID-19 vaccination might have a protective effect against MIS-C (84), and vaccination can be safely administered to children who have previously suffered from MIS-C (85). Even if MIS-C is associated with high ICU admission rates and organ involvement (mostly cardiac), the prognosis is generally good and most children recover rapidly, usually already during hospitalization (86).

A further complication of SARS-CoV-2 infections in children is post-acute sequelae, or “long COVID.” Systematic reviews show that most studies estimating the prevalence of long COVID in children have substantial limitations and that, even when there are control groups, it is difficult to differentiate between post-infectious long COVID and other pandemic-associated symptoms (11, 87, 88). The major limitations include a lack of a clear case definition for long COVID, arbitrary length of follow-up, inclusion of non-laboratory confirmed infections, and low response rates. Furthermore, there is potential for recruitment bias when people with a history of adverse effects could be more likely to accept recruitment in studies of self-reported symptoms (88). A less discussed topic, nocebo effect as seen with other exposures (89, 90), may also warrant further study in the context of long COVID.

A Norwegian report on healthcare use reported that SARS-CoV-2 infection in children had little impact on healthcare usage during the subsequent 6 months after the infection (91). A Danish cohort study concluded that long COVID in children is rare and mostly resolves after a short duration (92). Persisting symptoms have been reported after infections with other viruses. For example, for Epstein–Barr Virus, persisting symptoms, such as fatigue have been reported in up to 20% (93), and a large cohort study found that persisting symptoms in adolescents were similar after SARS-CoV-2 or influenza infection (94). One of the important outcomes of the pandemic is thus, arguably, to have raised awareness for long-term sequelae from infections more broadly and the associated unmet therapeutic need.

### 3.4. Co-morbidities associated with severe COVID-19

It is important to identify co-morbidities which increase the risk of severe COVID-19 (95), as children with these co-morbidities may benefit more from individual protective measures. An early systematic review identified that obesity increased the risk of severe COVID-19 (relative risk ratio 2.87) (96) and cross-sectional studies suggest that diabetes type 1 and congenital heart disease also increases the risk (97). A meta-analysis concluded that children with neurological and cardiac co-morbidities, as well as obesity, have an increased risk of ICU admission or death (98). Further systematic reviews identified chronic lung diseases and prematurity (99) as well as obesity, congenital heart, chronic lung and neurological diseases as risk factors for severe COVID-19 (100).

A study from Switzerland reported that co-morbidities were associated with a higher rate of hospitalization but not of ICU admission (6). In Germany, excluding children without co-morbidities decreased the incidence of ICU admission from 0.22 to 0.09 per 100,000 children (101). In the UK, more than 60% of children with MIS-C were reported to have co-morbidities, and from the eight children that died, all had co-morbidities (102). Also in the UK, 76% of the pediatric COVID-19-associated deaths during the first year of the pandemic were children with co-morbidities (103).

### 3.5. Effect of different SARS-CoV-2 variants

The main waves in Europe can be crudely classified into the first 2020 wave of the predominantly D614G variant of the Wuhan reference type (104, 105); the second wave in autumn and winter of 2020 to 2021, which included also Alpha (106), estimated to be approximately up to 30 to 50% more infectious and lethal; the 2021 summer wave, which was mainly driven by the even more infectious and lethal Delta variant (107, 108); and finally, the winter wave of 2021 to 2022, which was mainly driven by the Omicron variant which was less virulent at least partly due to high disease-relevant cellular immunity in the population at the time of its arrival (109), but substantially more infectious due to transmission-relevant antibody immune evasion (110, 111).

A question commonly raised was whether a new variant caused more severe disease in children compared with previous variants. For each new variant (Alpha, Delta and Omicron), it was initially suggested in media and early reports that the variant would be more severe in children (112, 113). Many such early data was based on limited sampling of children, and when taking into account more robust measures of relative virulence there was no indication that any of the variants had a disproportional virulence toward children, which would also require a special biological mechanism related to the mutations (113–116).

## 4. Basic immunology and pathology of pediatric COVID-19

In the following chapter, we provide a succinct overview of the variations in immunology and pathology of COVID-19 between children and adults relevant to the topic.

### 4.1. Innate immunity

While much remains to be understood, it is now clear that compared with adults, children react differently to SARS-CoV-2 on several levels. Compared with adults, children have much higher numbers of innate immune cells in their nasal mucosa and express higher levels of the major pattern recognition receptors involved in recognizing SARS-CoV-2 (117). The activation of these pattern recognition receptors results in the production of type I and III interferons, which helps to control SARS-CoV-2. Interferon production after SARS-CoV-2 infection happens faster in children (118) and children infected with SARS-CoV-2 have higher levels of certain cytokines and chemokines in their nasal fluid (119).

In blood, children infected with SARS-CoV-2 have an early expression of genes associated with interferon production and a reduction of myeloid cells, dendritic cells, natural killer cells and classical (CD14 + CD16-), intermediate (CD14 + CD16+) and non-classical (CD14-CD16+) monocytes (117, 119–122). Adults with COVID-19, especially severe COVID-19, also have low levels of dendritic and natural killer cells, but only show a decrease of non-classical monocytes while classical and intermediate monocytes, which are involved in the cytokine storm, increase (120, 123–125). Furthermore, during early disease, children have higher levels of C-X-C motif chemokine ligand 10 (CXCL10), granulocyte-macrophage colony-stimulating factor (GM-CSF), and interleukin (IL)-17A, while tumor necrosis factor (TNF) and IL-6 levels are similar when compared with adults (43, 122, 126).

### 4.2. Adaptive immunity

Levels of SARS-CoV-2-specific immunoglobulin (Ig) A and IgG in nasal fluid have mostly been reported to be similar in children and adults (119). Rapid and coordinated appearance of SARS-CoV-2-specific CD4+ and CD8+ T cells in blood is associated with faster clearance of SARS-CoV-2 (127). Children with COVID-19 have higher lymphocyte counts, with a higher proportion of innate lymphoid and non-clonally expanded naïve T cells (128, 129). They also have higher numbers of T follicular helper cells, which are important for an early antibody response (130). Furthermore, they have lower T cell responses S and ORF1 proteins and reduced CD4+ T cell effector memory (43, 119, 128, 130). Results of T cell responses against nucleocapsid (N) and membrane proteins are conflicting with some studies showing lower (130) and others higher (43, 119, 128) levels in children.

In children with COVID-19, genes associated with B cell activation are expressed earlier than in adults (122). Studies comparing levels of SARS-CoV-2-specific antibodies between children and adults report conflicting results. Compared with adults, an early rise in specific antibodies (123, 125) has been observed in children. Some studies have also reported that children, although having milder infections, mount higher antibody levels which persist for longer (50, 131). However, there are also studies reporting that children are less likely to seroconvert to SARS-CoV-2 compared to adults (44, 45), and have lower specific neutralizing antibody levels (43, 132). The role of endemic coronavirus infections in inducing pre-existing cross-immunity protection against SARS-CoV-2 is debated (133). Healthy children mostly possess SARS-CoV-2 IgM, suggesting that children have less-experienced but more polyreactive humoral immunity compared to adults (134). When interpreting these results, it is important to consider that antibody levels depend on the disease severity, the timing of measurement after exposure (135–137), the type of antibody (IgA, IgM, IgG), and the target of the antibody measured, e.g., spike (S) protein, nucleocapsid (N) protein, non-structural proteins (NSP) or open reading frames (ORF) (138). Many factors, including different affinity and distribution of angiotensin converting enzyme 2 receptors, differences in vitamin D and melatonin levels, have been suggested to explain the age-gradient of severity in COVID-19 (139). However, apart from the above-described differences in the immune system, the most likely explanation is the overall low observed prevalence of pre-existing endothelial injury and subsequent coagulation activation in the pediatric population (140–142).

## 5. School policies in Europe

In the following chapter, we present a comprehensive overview of the various migration measures implemented in different European countries to control the spread of COVID-19. These measures include but are not limited to school closures, regular testing, face mask mandates, and ventilation improvements, along with other non-pharmaceutical interventions. Additionally, we meticulously evaluate the uncertainties and comparability of data to ensure the accuracy and reliability of our findings.

### 5.1. Overview of applied mitigation measures

Strategies to prevent SARS-CoV-2 transmission in open schools can be divided into the following categories: (1) social distancing measures that reduce contacts, e.g., alternating classes or reduced class sizes; (2) measures that might render contacts safer, e.g., use of face masks, hand hygiene, or ventilation; (3) surveillance and response strategies, e.g., screening or testing strategies, and isolation protocols; (4) combined measures that take several of the above-mentioned approaches. In addition, vaccination can be regarded as an additional measure, which can be combined with others (143, 144). Although efficacy of vaccines toward preventing infections with new variants decreased rapidly due to antigenic drift (145), observational studies indicate that they remain effective in the protection against severe disease (146). Teachers have been a priority group for COVID-19 vaccination in countries such as Germany or Spain, achieving a high coverage rate in these countries (147). This was considered essential for Public Health authorities of some countries to keep schools open.

Public health policies and strategies, including restrictive measures, to control COVID-19 that were applied in European schools are summarized in Table 1. They include physical distancing, use of face masks, frequent hand-washing and use of hand sanitizer, cleaning and disinfection, and ventilation and respiratory etiquette. European countries have been reinforcing and modifying sanitation protocols throughout the pandemic to keep schools open. In Sweden, pre-schools and schools for children up to 16 years of age stayed open throughout the first wave, and as in Denmark and Norway, masks were not used in school settings during this or any later wave (148).

Bubble group structures (small group of individuals who limit their social interactions to only each other) within schools were implemented in some countries, for example Spain (149). Other countries, such as Norway, designed a traffic light-monitoring system strategy to guide local school closures based on the rate of community transmission (150, 151). Levels were updated regularly from attending school in person to a hybrid model with a maximum attendance of 50% and social distancing measures outdoors.

European institutions such as the European Centre for Disease Prevention and Control declared that recommendations for COVID-19 in children and the role of school settings in transmission published in July 2021 were also valid for the following 2022–2023 academic year (152). In fact, the guidelines released during summer 2022 addressed the key messages of staying at home if unwell, maintaining a good standard of hand hygiene, adhering to good respiratory etiquette, ensuring good ventilation arrangements, and continuing to maintain high sanitation standards.

### 5.2. Discussion of uncertainties and comparability of data

**Figure 2:**
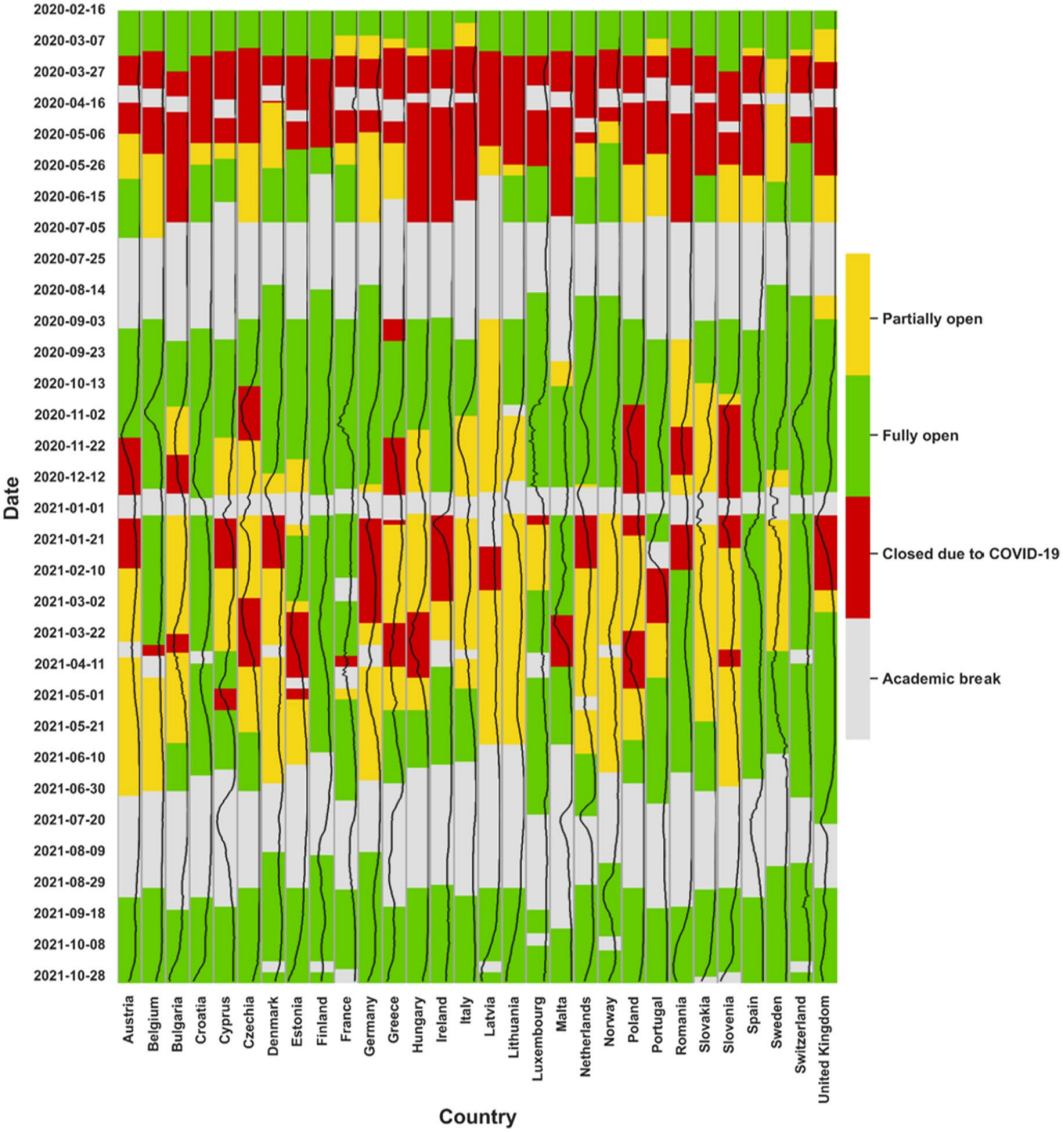
Closure of primary schools in European countries during the pandemic. The black line is showing the COVID-19 incidence per 10^5^ population for each country.

One of the most important limitations to direct comparison of the data is that European countries have faced different timelines and patterns of infections, lockdowns, school closures and re-openings during the pandemic (Table 1 and Figure 2). Further complicating comparisons, some areas or regions within each specific country often followed different guidelines, in accordance with the level of local community transmission (153).

Key epidemiological data for monitoring the COVID-19 pandemic in children, such as disaggregated ICU admission rates by age groups or hospital admissions or deaths due to COVID-19 (and not with an incidental positive test for SARS-CoV-2), have been difficult to obtain from some European countries. Also, official numbers may be differentially affected by testing, definition, and registration practices. With the aim to distinguish between children and adolescents who died as a result of SARS-CoV-2 infection from those who died of another cause but were incidentally infected with the virus, a UK review found a very low mortality rate of 2 per million (103), indicating the importance of separating these two types of data.

### 5.3. School closure

One of the earliest and most widespread actions taken by governments of European countries in 2020 was the closure of primary and secondary schools (Figure 2). This measure was applied because of previous knowledge inferred from other respiratory viruses such as influenza, where children are considered to be important drivers for transmission (52, 154). Globally, more than 190 countries closed their schools completely or partially, putting more than 1.6 billion students (or 80% of enrolled learners worldwide) out of their classrooms (30, 155). The closure of schools was highly disruptive, and the appropriateness of such measures has been debated extensively (156). With the emergence of the Omicron variant, the decision to close schools was no longer the norm and most European countries re-opened schools (Figure 2). Hybrid models of school closures during the COVID-19 pandemic involve a combination of in-person and remote learning. These models have been used in many countries as a way to reduce the number of students in schools at any given time, while still providing some level of in-person instruction. In some hybrid models, students attend school on alternating days or weeks, while in others, students attend school for a portion of the day and learn remotely for the rest. The effectiveness of these models in reducing transmission of COVID-19 is still being evaluated.

The effect of school closures on reducing SARS-CoV-2 transmission was estimated in modeling studies to be high early in the pandemic (35). However, real-world data obtained after school re-openings across many countries showed only a modest impact of school opening and that students and teachers were more likely to be infected via household or community contacts than in schools (4, 17, 157, 158). Low transmission risk in schools was concluded for the second half of 2020 in Germany (159). In Norway, few outbreaks were seen with schools open even during the Alpha wave, despite no use of face masks, explained by social distancing measures and social settings outside school (58, 150, 151). Similar results were seen in Switzerland, even at the time of high community transmission in December (160), and in Finland (161). These conclusions also seemed to hold true during the spread of the Delta variant (5). The studies, including systematic reviews, add to the evidence that school transmission is likely driven by home and community transmission rather than self-propagated (17), and that school closures have limited effect on reducing transmission.

### 5.4. Testing

Another commonly used initiative during the pandemic has been to periodically test students and staff to prevent SARS-CoV-2 transmission within schools. The schedule for this testing varied in different settings, i.e., twice or 3-times weekly. In terms of testing, the UK applied a “test-to-stay” strategy, in which students were provided with rapid tests (162). Daily contact testing of school-based contacts was non-inferior to self-isolation for control of SARS-CoV-2 transmission, with similar rates of symptomatic infections among students and staff with both approaches (162). Some other initiatives, such as a school-sentinel-surveillance network in Catalonia (Spain), have been implemented to monitor and evaluate SARS-CoV-2 infections in schools. This project aimed to do diverse screening strategies in a cohort of students and staff to monitor acceptance and potential impact (163). In some countries such as Austria, on-site school-entry testing were applied to control school transmission (164).

Contact tracing strategies have changed throughout the pandemic. For example, initially, in France, when a case was detected, contact cases that tested negative continued in-person learning while positive contacts switched to distance learning as they isolated for 10 days. This changed when the Omicron variant emerged, requiring contact cases to self-test immediately and again on the second and fourth days without any isolation required (165). Similar policies were implemented in Spain with contacts allowed to continue in-person learning if asymptomatic (166).

Systematic reviews of contact tracing studies and population-based studies have been published to inform on the role of children in the transmission of SARS-CoV-2 in household or educational settings (17). In Denmark, the National Board of Health published a booklet for parents in September 2020 with specific recommendations for parents in regards to testing their children and day-care or school attendance (167).

Several studies have addressed potential roles of regular nasal or saliva rapid testing in school children and teachers, showing that these provide little or no benefits on the overall community SARS-CoV-2 burden (168), local transmission, or risk of hospitalizations for children (which remains extremely low) (169) or adults working in schools, compared with adults who do not (170).

The cost-effectiveness and feasibility of systematic antigenic testing of asymptomatic children or staff depend on the community infection levels (171). Self-administered swabs are less intrusive and may be better suited for children than mass testing at school locations. However, these benefits must be weighed against the potential loss of sensitivity if self-administered swabs are not used appropriately (172). Evidence of effect of routine asymptomatic mass testing in broad populations is conflicting and context-dependent (173–176), and there is little evidence of efficacy in school settings, where such testing practices could also be disruptive. For a long time, Denmark was the country with the highest number of PCR and antigen tests performed per capita, testing its population much more than neighboring Scandinavian countries but not with clear associated benefits (176). Therefore, testing should arguably be prioritized for symptomatic children, especially if they belong to a risk group (177). With the Omicron variant and mass vaccination lowering IFRs (79), benefits of reducing transmission toward vulnerable groups are further reduced and shift the trade-off further toward emphasis on focused contact tracing near vulnerable children.

### 5.5. Masking

Similar to transmission of influenza virus (178), the Cochrane review for SARS-CoV-2 indicates little and uncertain effect of masking on broad adult populations (179). These reviews emphasize random controlled trials and low-risk studies and thus put less emphasis on confounded observational studies. Data on the efficacy of masking children in school settings are limited, and no randomized control trial has been performed. Consistent with this low evidence level, debate on this mitigation was controversial during the pandemic, and policies differed substantially between countries. A notable difference was observed between policies of some larger European countries, notably Italy and Germany, aligning with views in the US, and some other European countries reluctant to apply masking in school settings, with, e.g., Norway, Denmark, and Sweden never applying masking to primary school children. The CDC and AAP recommended masking of children aged 2 years or more (180), whereas the WHO (181), UNICEF and other international organizations only recommended masking from 5 years of age, when the community SARS-CoV-2 transmission was high.

Evidence of mask efficacy in school settings is low or inconclusive, and the safety for children has been questioned (182). While there is evidence that high-quality masks can block SARS-CoV-2 transmission in strictly controlled settings, their efficacy does not automatically translate to real-life scenarios, especially for children. A recent large study from Catalonia (Spain) found no differences in SARS-CoV-2 cases in children of similar age groups attending classes with or without mask requirements (183), confirming previous studies (182). Conversely, studies from the US identified benefits from masking school children (184, 185), while in Finland no effect was identified (186), although these studies are at a lower evidence level than the Catalonia study due to ecological design and risk of bias from contemporary use of different non-pharmacological interventions.

Concerns also exist regarding possible negative effects of regular mask use on child health and development. Masks may impede communication (verbal and nonverbal) and understanding and transmission of emotions, as well as speech and language development (182). As every decision in medicine, masking requires balancing benefits and risks and balanced positions between extremes (mandatory universal masking vs. no masking at all), as well as strategic use of masking policies through personalized approaches (focusing on high risk population and/or periods of significant local transmission and burden on health systems) (187). We also note that a recent randomized clinical trial (188) found no differences between different types of masks (medical masks and N95 respirators) in preventing COVID-19 among health care workers who provided routine care to patients with COVID-19, which is a surprising result that may suggest an overall modest effect of the intervention, consistent with other randomized trials.

### 5.6. Ventilation and other non-pharmaceutical interventions

Other NPIs to mitigate the risk of acquiring SARS-CoV-2 in school settings have been proposed, albeit with only little evidence of efficacy exists to date. One Cochrane systematic review from early 2022 compiled published and unpublished research findings, largely from modeling studies, on different measures that could be implemented in schools (189). Some specific studies are highlighted in more detail below.

A study from Germany assessed the efficiency of air purifiers in reducing aerosols in high-school classrooms (190). The authors estimated a reduction of virus-containing aerosols by a factor of six; however, no clinical endpoints were investigated, with the certainty of evidence graded as very low by the Cochrane review authors. Of note, air purifiers do not render ventilation redundant, but should be seen as a supplementary tool.

In a non-randomized study from Italy, the association between the rate of a mechanical ventilation system installation at schools and SARS-CoV-2 infection in the respective classes was investigated (191). Ventilation between 10 and 14 liters per second per student reduced the likelihood of infection by 80%.

A survey study from the US, reported that elementary schools with face masks and ventilation strategies in place had lower SARS-CoV-2 incidence rates (192). Ventilation strategies comprised opening windows or doors, the use of fans, or the combination thereof with air filtration, purification, or irradiation.

Several other similar studies that assessed combined measures were also evaluated in the Cochrane review, but all were categorized as very low certainty of evidence, with an inherent difficulty of disentangling mitigations to truly attribute effects to specific measures. It has been shown that portable HEPA air cleaners can reduce exposure to simulated SARS-CoV-2 aerosols in indoor environments, especially when combined with universal masking (193) and it is of interest whether this translates to school/classroom settings, which were not studied.

The ventilation aspect has also been addressed in national guidelines. The German consensus guideline on measures to prevent and contain SARS-CoV-2 transmission in schools recommends the implementation of ventilation strategies, while acknowledging that existing evidence has very low certainty (194). In addition, they recommended against using classrooms that have neither windows nor ventilation systems and concluded that mobile air purifiers should only be considered in exceptional situations.

Therefore, data about air purifiers suggest promising perspectives, but evidence is still preliminary and it will be important to generate better evidence to quantify possible benefits in school settings and, especially, feasibility in order to establish the cost-effectiveness, particularly in low-resource settings and in a post-pandemic high-immunity setting. As noted in a recent Lancet Task Force Commission review (195), improving building ventilation systems may carry benefits beyond protection from COVID-19, e.g., protection against other respiratory infections, and could be a wise cost-effective investment for population health in the longer term, if proved effective, especially in school settings (196).

## 6. Vaccination policies for children across Western Europe

On 21 December 2020, the European Medical Agency (EMA) approved the first vaccine against COVID-19 for adults and children over the age of 15, less than 1 year after the start of the pandemic. In May 2021, the vaccine was approved for 12–15-year-olds, 6 months later for 5–11-year-olds and, in February 2022, approval for children from the age of 6 months to 4 years was given. Currently, there are three approved pediatric vaccines in Europe, two of them (BNT162b2—Comirnaty^®^, mRNA-1273—Spikevax^®^) from 6 months of age and one (NVX-CoV2373—Nuvaxovid^®^) from 12 years of age (197).

**Figure 3:**
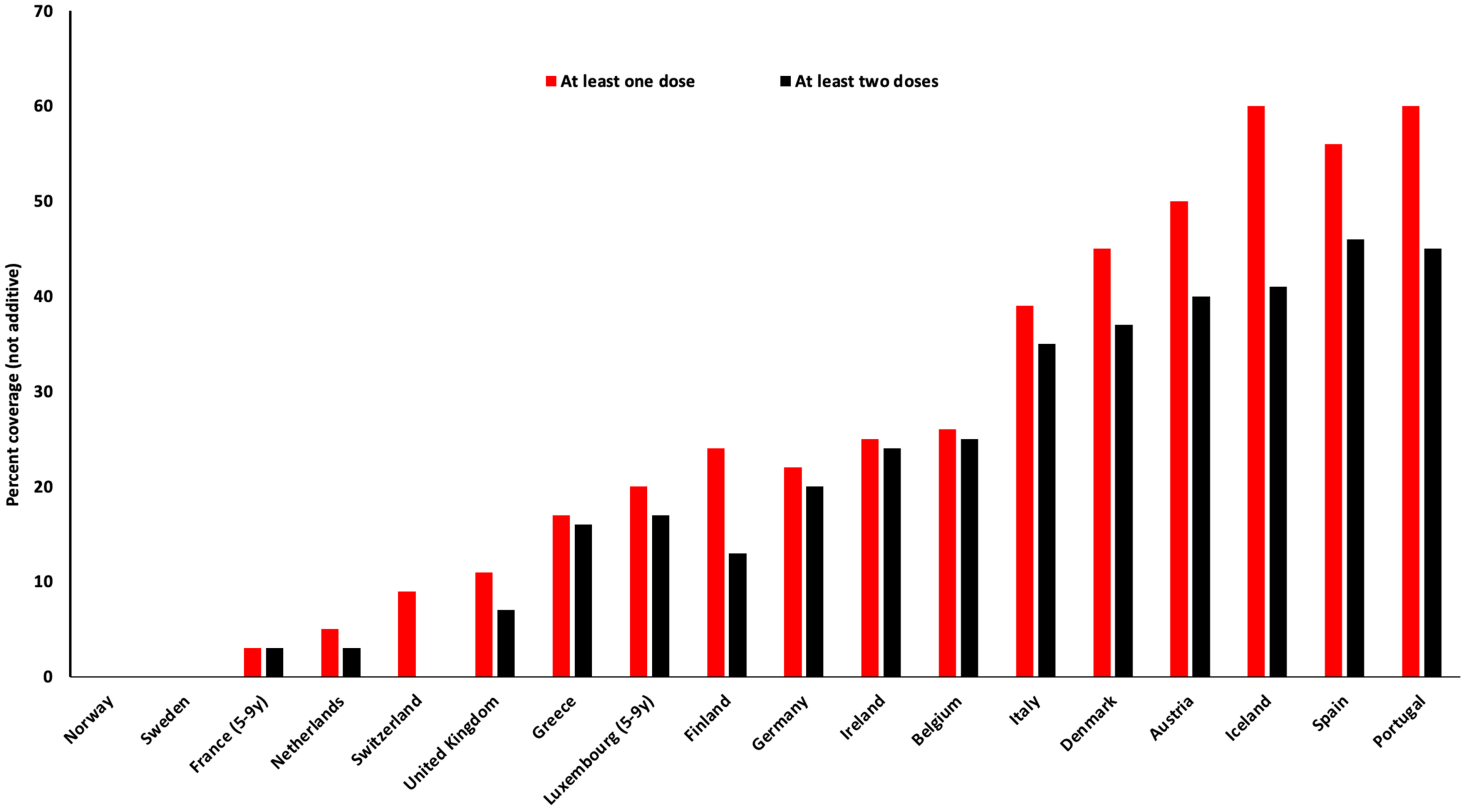
COVID vaccine doses per 100 children between 5 and 11 years of age (as per November 2022).

All European countries have published national COVID-19 vaccination policies, with marked differences between them. For example, some countries introduced vaccination in adolescents and children as soon as they were approved, whereas other countries postponed vaccinating children to further evaluate the available data. In the Nordic countries, Denmark and Iceland chose to recommend vaccination of all 5–12-year-olds, whereas Finland, Norway and Sweden only recommended vaccinating 5–11-year-olds at high-risk of severe disease or within high-risk households (Table 2). Most European countries have COVID-19 vaccine coverage rates well below 50% in 5–11-year-olds (Figure 3 and Table 2), which is remarkably low when compared to other routine childhood vaccines. Two European countries that had recommended vaccination for all children more than 5 years of age, discontinued vaccinations in under 18-year-olds, namely Denmark (June 2022) and the UK (November 2022), with the exception of high risk groups where the vaccine is offered, but not recommended. Many European countries continue vaccinating children and adolescents against COVID-19, although the majority do not recommend boosters. In the US, vaccination of all children from 6 months onwards has been recommended (198), with low uptake in toddlers, whereas no European country has recommended vaccinating all children under 5 years of age.

**Table 2:** SARS-CoV-2 vaccination policies and rates for different pediatric age strata, across European countries (as per November 2022).

Country	Ages	Start of vaccination	General vaccination stopped	At least one dose (%)	At least two doses (%)	Booster (%)
UK	5–11	Apr 2022	Nov 2022	11.2	6.7	0.1
	12–15	Sep 2021	Nov 2022	50.4	37.9	1.1
	16–17	Aug 2021	Nov 2022	64.2	50.9	13.9
IRE	5–11	Jan 2022	Ongoing	25	24	-
	12–15	Aug 2021	Ongoing	75	70.4	36.6
	16–17	Aug 2021	Ongoing		85.4	
GER	5–11	Dec 2021	Ongoing	22.4	19.9	-
	12–17	Aug 2021	Ongoing	74.4	69.5	31.4
NETH	5–11	Jan 2022	Ongoing	5	3	-
	12–17	June 2021	Ongoing	59	56	2
BEL	5–11	Nov 2021	Ongoing	26.2	24.7	0
	12–15	Sept 2021	Ongoing	72.7	71.7	15.2
	16–17	Sept 2021	Ongoing	83.6	82.6	29
LUX	5–9	Nov 2021	Ongoing	20	17	-
	10–14	June 2021	Ongoing	62	59	-
	15–19	June 2021	Ongoing	84	81	-
FRA	5–9	Dec 2021	Ongoing	3.4	2.8	-
	10–11	Dec 2021	Ongoing	10.1	8.4	0.5
	12–17	June 2021	Ongoing	83.6	81.1	19.3
DEN	5–11	Nov 2021	June 2022	44.5	36.9	-
	12–15	July 2021	June 2022	78.7	77.0	0.5
	16–19	May 2021	June 2022	88.4	87.3	46.4
NOR	5–11	–	–	–	–	–
	12–15	Sept 2021	Ongoing	86.4	13.5	–
	16–17	Sept 2021	Ongoing	98.1	88.1	–
SWE	5–11	–	–	–	–	–
	12–15	Oct 2021	Nov 2022	71.9	66.8	–
	16–17	Aug 2021	Nov 2022	80.3	75.8	–
ICE	5–11	Jan 2022	Ongoing	60	41	–
	12–15	Aug 2021	Ongoing	81	76	2
	16–29	June 2021	Ongoing	90	83	52
FIN	5–11	–	–	24.1	13.3	–
	12–17	Aug 2021	Ongoing	75.1	68.2	3.9
SPA	5–11	Dec 2021	Ongoing	55.8	46.0	–
	12–15	Aug 2021	Ongoing	92.5	96.1	–
	16–17	July 2021	Ongoing			
POR	5–11	Dec 2021	Ongoing	60	45	–
	12–17	Aug 2021	Ongoing	99	98	–
ITA	5–11	Dec 2021	Ongoing	38.5	35.3	–
	12–19	May 2021	Ongoing	86.6	83.9	46.6
SWI	5–11	Jan 2022	Ongoing	8.6	nr	nr
	12–15	May 2021	Ongoing	47.1	nr	nr
	16–17	Dec 2020	Ongoing	nr	nr	nr
AUS	5–11	Nov 2021	Ongoing	50.4	40.1	–
	12–15	May 2021	Ongoing	80.1	75.2	–
	16–17	May 2021	Ongoing	97.3	95.9	69.3
GRE	5–11	Dec 2021	Ongoing	17.4	15.5	–
	12–15	Oct 2021	Ongoing	36.3	33.2	–
	16–17	Aug 2021	Ongoing	58.9	54.3	–

nr, not reported.

Please replace by Vaccine by EU country: https://vaccinetracker.ecdc.europa.eu/public/extensions/COVID-19/vaccine-tracker.html#age-group-tab; UK, https://coronavirus.data.gov.uk/details/vaccinations?areaType=nation&areaName=England; Ireland, https://www.hpsc.ie/a-z/respiratory/coronavirus/novelcoronavirus/vaccination/covid-19vaccinationuptakereports/; Netherlands, https://www.rivm.nl/sites/default/files/2022-09/Deelname_COVID-19_vaccinatie_in_Nederland_20220905_1109_definitief.pdf; Germany, https://impfdashboard.de/en/; Belgium, https://covid-vaccinatie.be/en; Luxemburg, https://sante.public.lu/fr/espace-professionnel/recommandations/conseil-maladies-infectieuses/covid-19.html; France, https://www.santepubliquefrance.fr/dossiers/coronavirus-covid-19/coronavirus-chiffres-cles-et-evolution-de-la-covid-19-en-france-et-dans-le-monde; Denmark, https://covid19.ssi.dk/overvagningsdata/download-fil-med-vaccinationsdata; Norway, https://statistikk.fhi.no/sysvak/antall-vaksinerte?etter=diagnose&diagnose=COVID_19&fordeltPaa=alder&alder=2,3; Sweden, https://www.folkhalsomyndigheten.se/folkhalsorapportering-statistik/statistikdatabaser-och-visualisering/vaccinationsstatistik/statistik-for-vaccination-mot-covid-19/; Iceland, https://www.covid.is/statistical-information-on-vaccination; Finland, https://thl.fi/en/web/infectious-diseases-and-vaccinations/what-s-new/coronavirus-covid-19-latest-updates/situation-update-on-coronavirus/coronavirus-infections-in-schools. Spain, https://www.sanidad.gob.es/profesionales/saludPublica/ccayes/alertasActual/nCov/documentos/Informe_GIV_comunicacion_20221111.pdf; Portugal, https://covid19.min-saude.pt/wp-content/uploads/2022/11/DGS_boletim_20221107.pdf; Switzerland, https://www.covid19.admin.ch/en/vaccination/persons; Austria, https://www.health.gov.au/sites/default/files/2022-11/covid-19-vaccine-rollout-update-24-november-2022.pdf; Greece, https://eody.gov.gr/en/https://vaccinetracker.ecdc.europa.eu/public/extensions/COVID-19/vaccine-tracker.html#age-group-tab.

Effectiveness of COVID-19 vaccination is defined as its ability to prevent infection, symptomatic disease, hospitalization, ICU admission and mortality, as well as the possible prevention of long COVID and MIS-C. As mentioned previously, most of the children that fall seriously ill with COVID-19 have an underlying condition (199). Pediatric vaccination policies should, therefore, carefully balance benefits and risks of vaccinating children and adolescents. A Danish nationwide healthcare register study showed reduced risk of SARS-CoV-2 Delta variant infection in adolescents that had been vaccinated with BNT162b2 (200). An American study including 24 pediatric hospitals indicated that two doses of BNT162b2 vaccine might be protective against MIS-C in 12–18-year-olds (84). In this study, 95% of hospitalized children with MIS-C were unvaccinated, and only unvaccinated children with MIS-C required ICU admission. However, the incidence of MIS-C declined substantially with the introduction of the Delta variant, and the incidence rate of MIS-C per SARS-CoV-2 infection has been estimated to decline an additional 95% for the Omicron variant (201).

Since mRNA vaccination is believed to reduce the likelihood of COVID infection and its severity in children, it would be expected that vaccination might also reduce the risk of long COVID in this age group to the same extent. However, there is currently not enough data to confirm whether vaccination protects against long COVID in children. As previously mentioned, the definitions and prevalence estimates of long COVID are highly variable and subject to substantial limitations (11). Because of this lack of clarity, it is even harder to assess the extent to which vaccination may reduce consequences of long COVID in children and this may complicate decision making (199, 202).

However, it is also important to take into consideration the effect of pediatric vaccination on transmission and the advantage that this might create in controlling the spread of infection. During the Delta variant period, mRNA vaccines appeared capable of reducing onward transmission (203). Vaccination protects against disease and also, to some smaller extent, against Omicron transmission in children (204), but due to the much larger immune escape (110, 145), vaccine effectiveness against Omicron transmission wanes within a few months (205, 206). A case–control study showed that primary immunization with two vaccine doses provided limited protection against symptomatic omicron infection; receiving a mRNA booster substantially increased protection, but that protection again waned with time (207). These findings make vaccination as a means of Omicron transmission control much more questionable, perhaps especially, due to the lack of clear evidence for school to community transmission discussed above.

Not all European countries have introduced a booster dose in children and adolescents (Table 2) and all countries refrained from providing a second booster. This might be explained by the frequently mild disease in this age group and the high prevalence of infection-induced immunity, leading to favoring vaccine administration in other age groups at greater risk of serious disease. A recent risk–benefit analysis also suggested that boosters to adolescents might be associated with net harm and that booster vaccine mandates are unethical (208); however this trade-off is still debated and seems to be conceived differently in the US and in Europe.

COVID-19 vaccines for children are, for the vast majority of cases, safe: the most common adverse events, such as injection site reactions, nausea and vomiting, are usually mild to moderate (197). A more serious adverse event associated with mRNA vaccination, diagnosed mainly in male adolescents, is myocarditis and pericarditis, a significant concern in the pediatric population, particularly in 16–17-year-old males. It can occur after the first dose of an mRNA vaccine, but the highest risk has been shown to occur within 14 days of the second dose (209). A Nordic study found up to 28 excess cases of myocarditis or pericarditis per 100,000 young men (16–24 years) that received the second dose of the mRNA-1,273 vaccine, and up to 6 excess cases per 100,000 of those vaccinated with the BNT162b2 vaccine (210), making these events very rare but still important to monitor and consider in relevant trade-offs moving forward. Furthermore, vaccines can have non-specific effects (positive or negative) on overall health (211), but it has not yet been investigated whether COVID-19 vaccines have important non-specific effects, for example benefits on other outcomes, or some longer term negative effects. Another debate relates to whether vaccination itself can cause MIS-C. Although rare, there have been a few published case reports, suggesting that additional surveillance is needed (212–214).

## 7. Trade-offs

In the upcoming chapter, we aim to identify the primary goals of mitigation measures, assess their effectiveness, and evaluate their unintended consequences and trade-offs. Our analysis will also delve into the distributional impacts of these measures on children, with careful consideration of their potential implications.

### 7.1. School closure

School closure has many adverse effects: (1) it represents a large educational loss and the associated longer-term economical implications (3, 20); (2) it aggravates social inequalities (21); (3) it impairs non-educational benefits of schooling, such as social life and monitoring of child mental health (23, 215–217); (4) it may lead to worse nutrition status and increased inactivity and obesity, in itself are risk factors for disease, thus potentially also worsening physical health (23, 218).

Even in countries with excellent remote learning options such as the Netherlands, school closure led to learning loss (3), whereas in Sweden, which did not close schools during the pandemic, no learning loss was reported (219). For many children, schools offer the main way to monitor mental health and school closures disrupted this informed support (215, 216).

A huge trade-off that comes with school closures pertains to the detrimental consequences for children and adolescents, both on individual and societal levels (23, 220, 221). In some places, suicide rates among adolescents were higher during the COVID-19 pandemic than in the pre-pandemic era (222). Moreover, anxiety and depressive symptoms were reported to increase in association with school closures during lockdown phases (221). Other consequences including lost school-driven monitoring of child health (223), access to school-supplied nutrition and sanitation and increase in domestic violence (224). All of these negative consequences had an disproportionately higher impact on females (20).

A Cochrane Review has aimed to map the evidence on unintended health and societal consequences of school-based measures to prevent and control the spread of SARS-CoV-2 (144). The authors identified a wide range of unintended consequences of school-based measures designed to prevent the spread of SARS-CoV-2, including psychosocial consequences (e.g., mental health issues, depression, loneliness) and equity and equality consequences (e.g., disadvantages for children from low-income households, unfair distribution of work between genders for parents). However, the evidence base was small, with large gaps where more research is needed. The authors recommended that future research look at interventions such as fixed groups, alternating physical presence at school and staggered arrival departure and break times, as well as testing and quarantine measures.

When combining the adverse effects with the typically mild impact of disease on children vs. adults and most studies suggesting a limited tendency of school infection to drive community infection compared to the opposite (4, 16, 56, 58, 189, 221, 225) the school closures during the SARS-CoV-2 pandemic can be viewed now quite clearly as a mistake (223). Keeping schools open is now a top priority (19), and emphasis has instead moved to other mitigations in open schools to possibly combat transmission, as discussed further below.

### 7.2. Social distancing in school settings

Mitigations against transmission of infectious disease among children need to be evaluated on a background of benefit-harm trade-offs in two different scenarios; (1) mitigations applied to protect the children themselves from disease, or (2) mitigations applied to protect vulnerable groups in society from pediatric transmission chains (school-to-community transmission).

The most relevant social distancing measures during open schools are distancing of students in the class-room, reduced class sizes, alternate schedules, outdoor activities, the creation of social bubbles or playing groups, and general social distancing advice. While most of these social distancing measures can be said to have few adverse or negative effects, they are likely to affect transmission if contacts are reduced. However, even such social distancing measures may become disruptive over longer time periods and thus require trade-off considerations, especially in highly immune societies with low IFRs (79).

### 7.3. Masks

The effectiveness of masks in typical adult community settings may vary, depending on overall community transmission levels, societal context, and mask type. However, two randomized controlled trials on mask use suggest that the effect size may range from 0 to 20% (226–228), with small and uncertain effects concluded in the latest Cochrane review (179). In contrast, primary school children of 5–6 years age do not seem to benefit from mask use in the best available evidence so far from the Catalonian study (183). This study was not a randomized control trial, but a (cleverly) designed study using the monitoring of two class levels with and without mask use in close comparison, therefore better evidence may arise. The two results, when taken together, are consistent with the following possibilities: first, community transmission driving infection into schools rather than the opposite, resulting in little impact of school-based mitigation measures due to community infection; second, children having social contact patterns that impede the effectiveness of masks, or third, children lacking the ability to use masks adequately. Although community masking of adults and vulnerable populations is likely beneficial during periods of high transmission, the efficacy of masking school children remains uncertain, and may even pose potential risks.

### 7.4. Testing

Testing can be used to inform on transmission levels in a context of interest, but the information can also potentially be used in mitigation, by quarantine and follow-up contact tracing, to reduce social contacts between infected and non-infected. We can distinguish two general types of testing mitigations: symptomatic contact tracing that tests children who are known or suspected contacts of primary cases, and broad asymptomatic routine testing that seeks to identify asymptomatic index cases without knowledge of their contacts. The last mitigation affects more children, is more costly, and is also overall more disruptive, but can, in principle, identify cases in their pre-symptomatic state to reduce transmission chains.

Most countries in Europe applied a combination of these two strategies in schools, but with large variation in the degree to which these testing practices were mandatory. For example, in Denmark, for a short period in late 2021 and early 2022, children were recommended to be tested routinely as part of keeping schools open, but the testing was not enforced or required in order to attend school.

Contact testing to identify plausibly infected children may be useful and effective and can also, although sometimes overlooked, carry a substantial benefit in application toward vulnerable children (e.g., immuno-compromised children or other children at particular risk upon infection). Rapid antigen testing may provide a transmission protection around vulnerable individuals during periods of large community transmission, e.g., during winter, at a relatively smaller cost of testing for instance close contacts of those at-risk.

Broad asymptomatic testing may be useful for estimating infection levels in schools in order to inform decision making, but studies suggest that such testing regimes may not necessarily be very effective (176). Combining this with the possibility that these procedures may be disruptive to some children, balancing pros and cons would argue against applying such strategies, especially if mandated as a requirement for school attendance, which could be unethical given the debated absolute impact of being out of school.

### 7.5. Ventilation strategies

The current evidence for benefits of ventilation is of low quality, randomized controlled trials are lacking. However, a benefit of ventilation in class room settings is quite plausible, given that transmission is lower in outdoor settings (229, 230). Such benefits should be seen in context of other benefits of cleaner indoor air, and also of costs associated with implementing these mitigations compared to the same money spent elsewhere on public health, as they may require large infrastructure changes. However, in cases where improvements are not expensive, or simply likely to be cost-effective, such interventions should be explored much more actively primarily in high-risk settings such as long term care facilities.

## 8. Concluding remarks

Throughout the pandemic, disease severity of COVID-19 in children remained low. However, due to concerns that children in open schools could increase infection levels in the community and thereby endanger vulnerable and older people, mass mitigations remained applied to children also during later waves of the pandemic.

Evidence now suggests that school closures had vast consequences in terms of education loss, loss of school-related public health efforts, such as mental health monitoring, nutritional effects, and large impacts on social inequality, with these interventions disproportionately harming disadvantaged children and families. Careful consideration should be made in future pandemics from uncharacterized viruses, when decisions regarding possible school closures are to be made. As for other mitigation strategies, despite three pandemic years, large uncertainties remain both as to the effect and also negative effects of these mitigations, due to the absence of large randomized controlled trials for children in relevant settings. This absence of data represents a lost opportunity for evidence-based pediatric epidemiology.

These uncertainties have been reflected in the large variation in policies directed toward schools and children in Europe, much more distinct than for adults, with, e.g., smaller Scandinavian societies emphasizing a higher level of child normalcy (no mandates for school attendance or masks in primary school), and some larger countries emphasizing more authoritarian epidemiological control strategies, including direct mandates for school attendance, mask use, and indirect policies also negatively affecting children (such as curfews). Understanding the causes of these variations and the non-trivial relationships to outcome was beyond the scope of this review but would be a topic for future studies.

With these uncertainties and the arrival of Omicron and mass vaccination further driving down the IFR to low levels (79), we argue that these mitigations should be used carefully in contexts of high immunity. We emphasize focused contact tracing near vulnerable children, structured public health care and social inequality efforts, as the most meaningful of such mitigations, given the uncertainty regarding benefits and trade-offs. It is crucial that these mitigations are further evaluated and assessed to inform future pandemic policy and avoid mistakes done during the COVID-19 era. Although we aimed to present a well-rounded perspective on the subject, it is important to note that our approach was not systematic, and there may be selective reporting and other biases that should be considered when interpreting our findings. To further investigate the effects of various measures on children, future studies should utilize more rigorous methodologies. Additionally, we stress the significance of applying evidence-based medicine principles when implementing public health measures, and encourage more efforts toward producing high-quality evidence to better comprehend the impact of SARS-COV-2 infection and measures on children.

Since SARS-CoV-2 will continue to circulate, it is important to continue research on the risk factors, diagnostics and treatment for severe infections and for long COVID.

## Author contributions

All authors contributed equally to the review conception, design, and writing of the manuscript, read and approved the final manuscript.

## Conflict of interest

The authors declare that the research was conducted in the absence of any commercial or financial relationships that could be construed as a potential conflict of interest.

## Publisher’s note

All claims expressed in this article are solely those of the authors and do not necessarily represent those of their affiliated organizations, or those of the publisher, the editors and the reviewers. Any product that may be evaluated in this article, or claim that may be made by its manufacturer, is not guaranteed or endorsed by the publisher.
